# Predictive Examination of Phonological Awareness Among Hebrew-Speaking Kindergarten Children

**DOI:** 10.3389/fpsyg.2019.01809

**Published:** 2019-08-28

**Authors:** Dorin Wasserstein, Orly Lipka

**Affiliations:** ^1^Department of Learning Disabilities, University of Haifa, Haifa, Israel; ^2^Edmond J. Safra Brain Research Center, University of Haifa, Haifa, Israel

**Keywords:** phonological awareness, kindergarten, development, early literacy, Hebrew

## Abstract

The purpose of this study was to examine the development of phonological awareness (PA) skills among Hebrew-speaking kindergarten children. Specifically, the study examines the effects of cognitive, early literacy, and language skills to PA among Hebrew-speaking children at the middle (Early K) and end (End K) of kindergarten, and the contribution of various literacy and cognitive skills measured from the early kindergarten stage to the subsequent development of PA. Participants were 41 native Hebrew-speaking children (28 boys), ages 5–6, who were recruited from two kindergarten classrooms. A battery of cognitive, early literacy, and language measures was administered and ten PA skills were examined extensively. The results demonstrated the rapid growth of PA skills from Early K to End K. The participants were significantly better at manipulations at the syllable level, as compared to phonemes or consonants. Furthermore, deletion of a final consonant was found to be easier for them than deletion of an initial consonant. This finding emphasizes the *body-coda* segmentation tendency, which characterizes the Hebrew language structure. Strong-moderate positive correlations were found between PA and both letter naming and executive functioning at Early K. A strong correlation between letter naming and PA was found at End K. Regression analyses demonstrated that letter naming and executive functioning at Early K were the most significant predictors of PA at Early K, and that letter naming was the most significant predictor at End K. These findings highlight both universal and language-specific features of phonological awareness.

## Introduction

It is widely agreed upon that children usually master the initial phases of phonological awareness (PA) as preschoolers (Goswami and Bryant, 1990), beginning with syllable awareness around the age of three to four, and followed by onset-rime awareness, which normally appears around the ages of four to five. PA is the ability to isolate, delete, or manipulate different sub-lexical units within a word, such as syllables, onset-rimes, body-coda, and phonemes. PA is conventionally defined as the understanding that spoken words can be divided into smaller components (Chard and Dickson, 1999; Share and Blum, 2005). After children are taught to read and write, phoneme awareness, the most advanced form of PA, develops. Though this is the typical course of development, at least in English, it does not occur spontaneously. As children grow older, they become increasingly sensitive to smaller and smaller parts of words, and therefore can manipulate them (Trehearne et al., 2003). This progression is widely viewed as cross-linguistic and universal (Anthony and Francis, 2005). However, the precise nature of the large-to-small-unit sequence and the levels of proficiency of speakers in different languages can vary. For example, several studies found that 5-to-6 year old preschool children who speak Turkish, French, or Italian tend to attain syllable awareness faster than children who speak French or English (Cossu et al., 1988; Demont and Gombert, 1996; Durgunoğlu and Öney, 1999), suggesting that PA is affected by the saliency of spoken units in different languages (Ziegler and Goswami, 2005; Verhoeven and Perfetti, 2017).

### PA in Different Languages

Most of the studies examining the development and the contribution of PA to literacy skills have been conducted in the English language (Share, 2008). This, understandably, means that that development of theories of PA and reading, as well as instructional approaches and remediation methods, is based on the unique characteristics of the English language. But English, according to Share (2008), is an outlier orthography among European alphabets in terms of spelling-sound correspondence. As such, the role of PA in predicting reading skills is likely to vary between English and other orthographies. More precisely, transparent European alphabets, orthographies with a one to-one mapping between letters and sounds, such as Italian, German, or Finnish, should naturally promote high levels of PA while creating fewer obstacles for most readers, leading PA to be a weaker predictor of reading development. In contrast, in inconsistent orthographies such as English, where many letters can be pronounced in multiple ways, PA is more likely to be a central component in reading development and a stronger predictor of reading skills (National Reading Panel, 2000; Ziegler and Goswami, 2005; Share, 2008; Melby-Lervåg et al., 2012). For example, Furnes and Samuelsson (2010) compared associations between PA and reading skills among English-speaking and Scandinavian children. The study showed that PA contributed to the identification of poor readers in English in both the first and second grades, while in Norwegian/Swedish children, PA was related to reading difficulties only in first grade.

A group of studies demonstrate that PA has similar predictive power across languages, regardless of transparency (e.g., Furnes and Samuelsson, 2009; Caravolas et al., 2012). However, other studies demonstrate that in transparent languages, such as Dutch, Latvian, and Finnish, the predictive power of PA is limited to the early phase of formal reading instruction in Grade 1 (e.g., Holopainen et al., 2000; Dufva et al., 2001; Sprugevica and Høien, 2003). Meanwhile, in deep orthographies like English, where there is need for more time to learn to read, PA skills predict reading skill longer than in more shallow orthographies (e.g., Wagner et al., 1997; Cardoso-Martins and Pennington, 2004; Parrila et al., 2004). This unreasoned issue can be clarified with additional data from non-European languages, in which most readers learn to read.

The present study examines PA, in a non-European language that uses a non-alphabetic script – Semitic Hebrew. It is important to note that while Hebrew is not one of the major languages spoken worldwide (spoken by only 10 million people), it is one of the Semitic languages, like Arabic, which is the native language of hundreds of millions of people. Moreover, the Abjad system, which was developed by Semitic language speakers, constitutes a part of a writing system used by billions (Daniels and Bright, 1996). The many offspring of the original Semitic Abjad include today’s Semitic (e.g., Hebrew, Arabic) and non-Semitic Abjads (e.g., Urdu, Malay), and all European alphabets originating thereof, with the Greeks’ borrowing the Semitic (Phoenician) Abjad.

### PA in Hebrew

A relatively small number of longitudinal/predictive and training studies have examined the role of PA in Hebrew (Bentin and Leshem, 1993; Kozminsky and Kozminsky, 1995; Lapidot et al., 1995; Shatil and Share, 2003; Nevo and Breznitz, 2011). These studies have shown that training in PA skills during kindergarten later led to improved reading abilities. Further work has explored precisely which phonological units are the crucial ones in Hebrew. Focusing on preschool and kindergarten, studies demonstrate that in Hebrew CV (body) units (*not* rimes, as in English) are more accessible than isolated phonemes among native Hebrew speakers (Bentin et al., 1990; Ben-Dror et al., 1995; Share and Blum, 2005; Share, 2018). In addition, access to single (consonant) phonemes has been found to shift from an early pre-literacy advantage for initial phonemes to a literacy-engendered preference for final phonemes (codas) (Share and Blum, 2005; Saiegh-Haddad, 2007). Furthermore, when comparing PA among 5 years old native speakers of Spanish, Hebrew, and Cantonese, a significant advantage emerged in final phoneme isolation for the Hebrew speakers and there were no significant differences between the three languages in initial phoneme (consonantal) isolation (Tolchinsky et al., 2012).

Correlation studies in Hebrew reported weaker correlations between end-of-Grade 1 reading and PA compared to English (Share and Levin, 1999). When preschool and kindergarten were examined longitudinally, there was a strong correlation between Hebrew reading and PA in the *middle* of Grade 1, when most children are still learning basic letter-sound correspondences (Bentin and Leshem, 1993). This phenomenon was conceptualized by Share (2008) as the “functional opacity hypothesis”: “the PA-reading association is strongest when the spelling-sound code is opaque owing to intrinsic spelling-sound irregularities in the orthography or to incomplete mastery of a regular code.” This implies that the intrinsic relationship between PA and reading may be equally strong in both languages, with the only difference being one of timing (Share, 2018).

Taken together, this important role of PA is also reflected in the Israeli kindergarten curriculum (Ministry of Education, 2007), which refers to PA as a significant and important component required for the acquisition process of reading and writing in Hebrew.

Despite the recognition of its importance, no Hebrew-language study of PA has studied PA *developmentally*, that is, following the same children’s PA growth across time. All studies of PA to date have either been cross-sectional (e.g., Share and Blum, 2005) or, if longitudinal, have measured PA at only single time-point, with the aim of Time-1 to Time-2 prediction (e.g., Shatil and Share, 2003). The first aim of the present study is to begin filling in this gap by following children’s PA development over the course of the Kindergarten year which, in Israel, is the final year of pre-school education, preceding the commencement of formal reading instruction in Grade 1. Furthermore, more work is needed to clarify the nature of the relevant phonological units and the role of PA in the development of literacy in Semitic Hebrew.

There is an extensive research showing clear links between specific variables and PA (Aram and Levin, 2001; National Early Literacy Panel [NELP], 2008; Castles et al., 2009; Lerner and Lonigan, 2016). However, little attention has been paid to the development of the relationships between PA and early literacy and cognitive variables. Therefore, this study will also examine the developmental relationships between PA and early literacy and cognitive skills in Hebrew.

### Phonological Awareness and Letter Knowledge

Letter-learning plays a crucial role in the development of phonemic awareness.

Specifically, knowledge of letter sounds and access to phonemic representations of speech are the two critical foundations for learning to read.

A study examining the relationship between PA and letter knowledge reported a bidirectional relationship among pre-reading 4- and 5-year-old English speakers, such that PA predicted growth in letter knowledge, and letter knowledge predicted growth in PA (Burgess and Lonigan, 1998). Furthermore, in a sample of 358 English speaking preschoolers, a bidirectional relationship between letter knowledge PA was found using growth models analyses (Lerner and Lonigan, 2016).

Similar results were found in a study on 100 children who speak Finnish (Lepola et al., 2005), which is reportedly the shallowest orthography in Europe (Seymour et al., 2003). In this study, the 5-year-old children’s letter knowledge was closely related to their PA (coefficient = 0.71). This indicates that in the Finnish language, which has a highly transparent orthography, kindergartners who can pick up and identify letters also perform better on phonological tasks requiring the analysis of larger (rhyme) and smaller (initial phoneme) segments of spoken words (Lepola et al., 2005).

### Phonological Awareness and Rapid Automatized Naming

Naming speed is the ability to rapidly retrieve the names of familiar items presented visually in a serial array (e.g., objects, colors, numbers or letters, or a combination of these in rapid alternating stimulus formats; Dencla and Rudel, 1974). The speed at which naming tasks are performed (or naming speed) is considered one of the strongest predictors of fluent reading (e.g., Wolf, 1984; Korhonen, 1995; Wolf and Bowers, 1999; Holopainen et al., 2001; Torppa et al., 2010; Papadopoulos et al., 2016). In the early stages of reading acquisition, previous studies have revealed positive associations between PA and Rapid Automatized Naming (RAN). Based on their longitudinal study, Lervåg et al. (2009) provided evidence of an earlier connection, 10 months before the start of formal reading instruction between phoneme awareness and non-alphanumeric RAN.

### Phonological Awareness and Vocabulary

Vocabulary skills have a significant impact on PA. One explanation for the relationships between PA and vocabulary is that phonological representations become more fully specified as breadth of vocabulary increases, to avoid confusion between similar sounding lexical items (Metsala, 1999).

Studies of both preschool (e.g., Chaney, 1992) and early elementary school children (e.g., Wagner et al., 1993, 1997) have demonstrated significant concurrent and longitudinal correlations between children’s vocabulary skills and their phonological sensitivity. The receptive and expressive language skills of typically developing 3-year-old children were found to predict concurrent scores on a composite PA measure (Chaney, 1992, 1994). In addition, improved PA has been linked to vocabulary growth in preschool children (e.g., Carroll et al., 2003; Lonigan, 2007). It is well established that vocabulary knowledge (typically assessed by receptive vocabulary tests) predicts variations in word recognition skills in reading (e.g., Stevenson et al., 1976). Dickinson et al. (2003), for example, found a correlation between PA and vocabulary among preschoolers. This finding was replicated by Hipfner-Boucher et al. (2014), who also found a moderate correlation between these two variables. It has also been proposed that vocabulary breadth promotes early development of PA (Metsala, 1999). In this context, longitudinal studies have shown that performance on receptive and expressive vocabulary tasks predicts later PA skills, among children between the ages of three and four in Finnish (Silvén et al., 2002) and among 4- to 6-years-old children in English (Ouellette and Haley, 2013; Hipfner-Boucher et al., 2014). Additionally, the link between vocabulary and PA has been conceptualized in the lexical restructuring model (LRM; Fowler, 1991; Metsala and Walley, 1998). According to this model, representations of words in the lexicon of very young children are holistic and gradually become more fine-grained and segmented during the preschool and early school-age years. Lexical restructuring is assumed to be a function of vocabulary growth that occurs in response to the learning of individual words within a spectrum of phonological similarity.

### Phonological Awareness and Executive Functions

Executive function (EF) is an umbrella term covering a set of cognitive abilities including attention, inhibition, working memory, and flexibility. These processes are necessary for academic functioning, as they enable the individual to control, monitor, and manage the performance of tasks (Cartwright, 2012). Both EF and PA have an important role in the development of reading skills (Cartwright, 2012). In fact, Bierman et al. (2008) found that EFs (working memory, inhibitory control, and shifting) predicted PA (blending and elision tasks) during the pre-kindergarten year. EFs were also found to contribute significantly to PA and orthographic knowledge among Hebrew-speaking kindergarten children (Shaul and Schwartz, 2014). These findings highlight the importance of examining cognitive, early literacy, language, and EF processes when examining PA development.

### Phonological Awareness and Working Memory

Working memory is a cognitive system that enables problem-solving through the retrieval of stored knowledge and its mental manipulation alongside newly received information (Swanson et al., 2008). According to the model proposed by Baddeley and Hitch (1974) and later elaborated by Baddeley (1986, 2000), working memory is a multicomponent system containing three constructs: the central executive, the episodic buffer, and the phonological loop.

The relationship between PA and working memory in general has been studied previously in students (e.g., Oakhill and Kyle, 2000). Ouellette and Sénéchal (2008), for example, found a moderate positive correlation between working memory and PA among 5-year-olds. The specific relationship between PA and the proposed phonological loop component of working memory, which is believed to maintain and processes phonological information (Baddeley, 1986), has also been addressed, with some studies positing that the two have a common underlying phonological processing construct (Siegel and Linder, 1984; Stanovich et al., 1984). In contrast, other studies have claimed to show that the two are distinct systems (e.g., Gathercole et al., 1991; Hecht et al., 2001; Alloway et al., 2004).

### The Hebrew Orthography

Hebrew is an Abjad or consonantal writing system (Daniels, 1990). The Hebrew orthography is a primarily consonantal alphabet that exists in both pointed and unpointed forms. Unpointed script is partly vowelized by means of four consonantal letters that function as vowels as well as consonants. Pointed Hebrew employs diacritical marks or points and is used in texts intended for young children, in poetry, and in sacred texts. The diacritical system provides a complete and virtually unambiguous representation of the vowels by means of small dots and dashes appearing mostly under, but sometimes above and between, the letters (Shatil et al., 2000). Children learn to read in pointed Hebrew, which has a near-perfect one-to-one grapheme–phoneme correspondence (Navon and Shimron, 1984). However, phoneme-to-grapheme relationships in both pointed and unpointed script are frequently variable, with a number of pairs of (once phonemically distinct) consonant letters now representing the same phoneme (Shatil et al., 2000). Most children attain proficiency in decoding pointed text within their first year of school (Share and Levin, 1999). Modern Hebrew has a rich inflectional morphology that permits considerable versatility in word order. Nouns and adjectives are marked linearly for number, near-arbitrary gender, and definiteness; verbs are marked for number, gender, and person; and they take infinitive, imperative, or indicative forms including past tense, future, and present or participial forms (Share, 2018).

### The Present Study

The main goal of the current study was to examine the development of PA during kindergarten in Hebrew, a transparent orthography. Kindergarten is an important stage in terms of PA skills development, as PA skill play a significant role in the acquisition of reading and writing abilities. Therefore, our main objective was to explore whether we would see an increase in PA during kindergarten. We hypothesized that there would be a significant difference between PA performance at the early kindergarten stage and PA performance at the end of kindergarten. Our second objective was to determine which PA skills develop at the middle and the end of kindergarten in the Hebrew language. The hypothesis was that similar to the PA development trajectory (Anthony and Francis, 2005), in the Hebrew language, we expected greater success on tasks requiring manipulation at the syllable level, compared to a single phoneme or consonant. In addition, due to the structure of the Hebrew language and the natural tendency for body-coda segmentation (CV + C) (Ben-Dror et al., 1995; Share and Blum, 2005), we expected that manipulations of final consonants would be easier to perform than manipulations of initial consonants. The third goal was to determine which cognitive and language measures predicted PA at the Early stage and End of kindergarten. The developmental design provided a unique opportunity to examine this change in skills during the critical kindergarten year, before the formal teaching of reading and writing.

## Materials and Methods

### Participants

Participants included 41 Hebrew-speaking children (28 boys) who were recruited from two kindergarten classrooms. The first measurements were taken in December (early kindergarten – Early K) when the average age of the children was 66.4 months (*SD* = 3.84, range = 60–72 months), and the second measurements were taken in July (end of kindergarten, End K). The sample comprised typically developing children with an average non-verbal IQ (*M* = 108.51, *SD* = 12.83), from medium to high socio-economic backgrounds. The children had no neurological disorders and came from monolingual Hebrew-speaking homes. According to the teachers of both kindergarten classes, the Israeli Ministry of Education’s *Foundations of Reading and Writing* program was implemented, *Likrat Kri’a Ve’ktiva* (in Hebrew, Toward Reading and Writing).

### Measures

Phonological awareness measures were administered at Early K and End K, while literacy, language, and cognitive measures were administered at Early K only. Task order was counterbalanced.

*Letter naming task.* In the *letter naming task*, participants were asked to name 11 printed Hebrew letters (of the 22 letters in the Hebrew alphabet). The task was based on an earlier study by Share and Blum (2005). Cronbach’s alpha for this task was 0.92.

The *Vocabulary Production Test* (Tavor, 2008), a standardized Hebrew-language test for children between the ages of two and eight was employed to assess language skills. Different pictures were shown to participants, who then had to answer questions in reference to the pictures, such as “What is this?”, “What is the child doing?”, and “How is the child feeling?” A standardized score was calculated for each participant based on the number of correct answers provided.

*Rapid serial naming* (Shatil, 1995). Participants were asked to name, as quickly as possible, 21 pictures of objects (e.g., house, dog, tree) and 21 names of colors (e.g., red, green, yellow). In each test, five different stimuli were repeated several times. Total naming time and number of errors were recorded for each test.

*Working memory: The Children’s Size Ordering Task* (CSOT; McInerney et al., 2005). In each trial, participants listened to a series of words and were asked to recall the list of words according to the physical size of the objects represented (e.g., “bird, earring, door” → “earring, bird, door”). Two practice trials were conducted, in which each participant performed two trials, each comprising two words. The number of words in each trial was then increased by one every two trials (maximum five words). The task was stopped when a participant failed to recall both trials with the same number of words.

*Executive functioning: Head–Toes–Knees–Shoulders task* (HTKS; Ponitz et al., 2009). This task examines self-regulation, listening comprehension, memory of instructions, attention, inhibition, and cognitive flexibility. It includes 20 items, each of which can receive a score of 0 (incorrect response), 1 (self-correction), or 2 (correct response). In the first part, participants are asked to place their hands on a certain part of their body in accordance with the instructions. In the second part, they are asked to put their hands on a part of the body other than the body part mentioned by the instructor. For instance, if a child was asked to put his hands on his shoulders, he should put them on his feet, and if he was asked to put his hands on his head, he should put them on his knees.

*Phonological awareness measure*. A child-friendly tool was developed for the present study specifically to assess the PA of Hebrew-speaking kindergarten children. The tool included 10 tasks, several of which were based on tasks developed by Tadmor-Troyansky (in press) in her study of the role of consonant versus vowel phonemes in Hebrew literacy learning. All 10 tasks assessed the ability to manipulate sub-lexical phonological units at the level of whole syllables, sub-syllabic multi-phonemic units, individual consonants, and vowels. Stimuli included both monosyllabic and bi-syllabic words, open and closed syllables, and syllables with simple (CVC) and complex onsets (CCVC).

All PA tasks were presented within short stories about four superheroes, each of which accompanied a different type of task (initial sound, final sound, segmentation, and synthesis). At the beginning of each task, a cardboard figure of a specific superhero was presented to the child, accompanied by a short story about the hero’s capabilities. The child was then asked to perform a task similar to that associated with the superhero (for example, “*Shon Rishon is an expert in initial sounds. With his magic wand, he is able to detect the first sound of every word he hears.*” “*Now, let’s see if you can act like Shon Rishon and tell me the first sound that you hear in a word.*”).

Each task consisted of two practice items followed by 10 test items. The words were spoken aloud by the instructor. Corrective feedback was given only on practice items. If the child gave a correct response, the following reply was given: “*That’s right! You’ve got the idea. Now let’s try another one.*” If the child gave an inaccurate response, the following reply was given: “*That’s not quite right. The first sound of this word is ________. Let’s try another one.*” and the task was discontinued after five consecutive errors. Each correct response received one point, with a maximum score for each phonological task of 10. All scores are presented as percentages of correct answers. To avoid fatigue and perseveration across tasks on a specific manipulation, the tasks were divided into two sessions and counterbalanced; Session 1 included initial sound and segmentation manipulations, and Session 2 included final sound and blending manipulations.

The ten PA tasks were as follows:

1. Initial consonant isolation in complex onset (consonants cluster) (CCVC) – participants were asked to say which sound they heard at the beginning of a sounded word. For example, “Say *kluv*. What is the sound at the beginning of the word *kluv*?” (i.e., /k/). Cronbach’s alpha for this task was 0.93.
2. Isolation of an initial consonant in simple onset from a CVCCVC word – participants were asked to say the smallest sound they heard at the beginning of a sounded word. For example, “Say *sargel*. What is the smallest sound at the beginning of the word *sargel*?” (i.e., /s/) Cronbach’s alpha for this task was 0.91.
3. Syllable segmentation in a CVCCVC word – participants were asked to segment a bi-syllabic word which included an open and a closed syllable, into syllables. For example, “Say/d
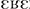
x/‘path’. Now, divide the word into syllables.” (i.e.,/de.rech.) Cronbach’s alpha for this task was 0.97.
4. Phoneme segmentation in CVC words – participants were asked to segment a sounded word into phonemes. For example, “Say *tik*. Now, segment the word into the smallest sounds you hear.” (i.e., /t/,/i/,/k/.) Cronbach’s alpha for this task was 0.93.
5. Deletion of an initial open syllable from a CVCVC word – for example, “Say *sadin*. Now, say *sadin* without *sa*. What’s left?” (i.e., *din*.) Cronbach’s alpha for this task was 0.97.
6. Deletion of an initial consonant from a CVC word – for example, “Say *zer*. Now, say *zer* without *z*. What’s left?” (i.e., *er*.). Cronbach’s alpha for this task was 0.82.
7. Phoneme synthesis – participants were asked to blend phonemes into a CVC word. For example, “The sounds are K-A-F. What word does this make?” (i.e., *kaf*.) Cronbach’s alpha for this task was 0.91.
8. Isolation of a final CVC syllable – participants were asked to say what syllable they heard at the end of a sounded word. For example, “Say *seret*. What is the sound at the end of the word *seret*?” (i.e., *ret*.) Cronbach’s alpha for this task was 0.93.
9. Isolating a final consonant – participants were asked to say the phoneme in a sounded word. For example, “Say *shaon*. What is the smallest sound at the end of the word *shaon*?” (i.e., *n*.) Cronbach’s alpha for this task was 0.96.
10. Deletion of a final consonant (simple coda) from a CVC unit – participants were asked to say a word without its final phoneme. For example, “Say *shem*. Now, say *shem* without *m*. What’s left?” (i.e., *she*.) Cronbach’s alpha for this task was 0.90.

In addition to scores for individual tasks, a general PA score was calculated by averaging scores across all ten PA tasks.

### Procedure

The tasks were administered at the middle and end of the kindergarten year (Early K and End K, respectively) with all children assessed at both time points. Only children whose parents returned a parental informed consent form participated in the study. Assessments were conducted individually in quiet rooms at their kindergartens. Three trained graduate students administered the tasks. To avoid fatigue, tasks were split into two separate sessions, each lasting between 20 and 30 min. For all tests, children were given examples and feedback before testing. PA skills were assessed at both the Early K and End K time. Literacy, language, and cognitive measures were administered at Early K only.

## Results

The first goal of the current study was to examine whether there was an increase in PA skills during kindergarten. Significant differences were found between success rates at the two time points for the general PA. The mean success rate of all the PA tasks at Early K (*M* = 29%) was significantly lower than the performance of the children at End K (*M* = 47%), *t*(40) = 9.45, *p* < 0.001. This demonstrates, as anticipated, that the kindergarten year, in Israel among Hebrew speakers, is a crucial time in the growth of PA knowledge.

**FIGURE 1:**
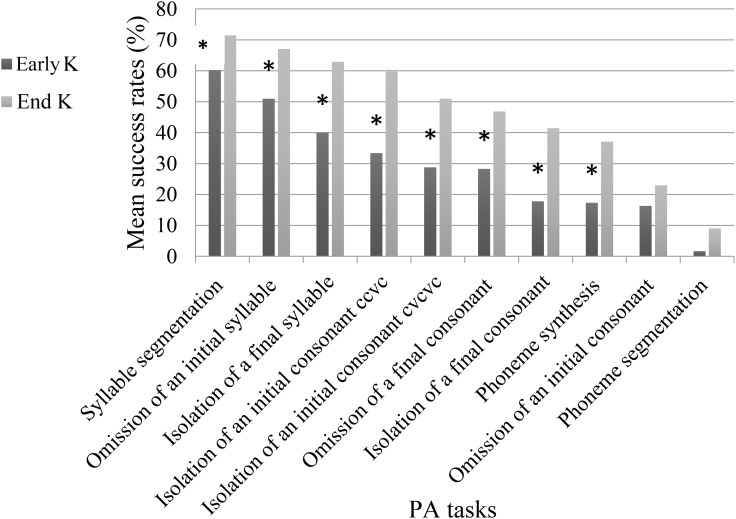
Developmental changes in success rates for each phonological awareness (PA) task between Early K and End K.

The second objective was to determine which Hebrew language PA skills develop at the middle and end of kindergarten. Figure 1 shows changes in the success rates for each of the ten PA tasks between Early K and End K.

A series of paired sample *t*-tests was conducted to shed light on the developmental sequence of the measured PA skills. As seen in Figure 1, at both time points, higher success rates were found for tasks requiring manipulation at the syllable level (*M* = 48) than for tasks requiring manipulation at the consonant and phoneme level (*M* = 29), *t*(40) = 6.457, *p* < 0.001. Moreover, the deletion of a final consonant (simple coda) from a CVC unit had a higher success rate (*M* = 37) than did the deletion of an initial consonant from a CVC word (*M* = 20, *SD* = 25), *t*(40) = 3.062, *p* < 0.005. No significant differences were found between the isolation of an initial consonant in simple onset from a CVCCVC word (*M* = 40, *SD* = 30.5) and the isolation of a final consonant (*M* = 30, *SD* = 33), *t*(40) = 1.963, *p* = 0.057. Also, a higher success rate was found for isolation of an initial consonant in simple onset from a CVCCVC word than for deletion of an initial consonant from a CVC word, *t*(40) = 3.347, *p* < 0.01. No significant difference was found between the isolation of a final consonant and the deletion of a final consonant (simple coda) from a CVC unit, *t*(40) = 1.722, *p* = 0.09. Additionally, phoneme synthesis had a higher success rate (*M* = 27, *SD* = 27) than did phoneme segmentation in CVC words (*M* = 5, *SD* = 12), *t*(40) = 5.151, *p* < 0.001, in which there appeared to be a floor effect. The latter was also found to have a lower success rate than deletion of consonants in general (*M* = 29, *SD* = 22), *t*(40) = 6.135, *p* < 0.001. Paired-samples *t*-tests were conducted for all of the ten PA tasks at Early K and End K. Results demonstrated that performance at End K differed significantly from performance at Early K for eight PA tasks. Only two tasks, syllable segmentation and deletion of an initial consonant, did not show significant differences between time points. Figure 1 presents success rates for each of the ten PA tasks at Early K and at End K.

Table 1 provides the means and standard deviations for the literacy and cognitive skills at Early K. These variables, color naming, object naming, vocabulary, working memory, and EF are further discussed with regard to their correlations with PA at both time points.

**TABLE 1:** Means (in percentages), standard deviations (in parentheses), for Kindergarten literacy and cognitive measures.

**Variables**	***M* (*SD*)**	**Min**	**Max**
Letter naming (SR)	0.34 (0.34)	0.00	1.00
Vocabulary (SR)	0.84 (0.06)	0.68	0.96
Object naming (RT)	32.83 (10.49)	17.00	59.00
Working memory (SR)	0.44 (0.14)	0.12	0.75
Executive functions (SR)	0.82 (0.15)	0.25	1.00
Total score PA Early K (SR)	0.29 (0.17)	0.00	0.64
Total score PA End K (SR)	0.46 (0.17)	0.13	0.86

*Success rates in percentages. Early K = middle of kindergarten, December. End K = end of kindergarten, June.*

Next, we examined which early literacy, language, and additional cognitive skills measured at Early K were correlated with PA skills at Early K and end End of K. Table 2 presents the correlation results.

**TABLE 2:** Correlations between early K PA, end K PA, naming speed, literacy, language, and cognitive measures (*N* = 41).

		**1**	**2**	**3**	**4**	**5**	**6**
1	Total PA early K						
2	Total PA end K	0.78^∗∗^					
3	Letter naming	0.41^∗∗^	0.63^∗∗^				
4	vocabulary	0.18	0.16	0.19			
5	Object naming	−0.06	−0.21	−0.36^∗^	0.04	0.62^∗∗^	
6	WM	0.25	0.23	0.18	−0.03	−0.19	
7	EF	0.41^∗∗^	0.29	0.19	0.30	0.18	0.22

*^∗^*p* < 0.05; ^∗∗^*p* < 0.0. Early K = middle of kindergarten, December. End K = end of kindergarten, June.*

At Early K, there was a moderate-strong positive correlation between general PA and EF [*r*_(__41__)_ = 0.41, *p* < 0.01] and between general PA and letter naming [*r*_(__41__)_ = 0.41, *p* < 0.01]. At the end of kindergarten, there was a strong positive correlation between PA and letter naming [*r*_(__41__)_ = 0.63, *p* < 0.01].

To determine which variables at Early K predicted PA at both time points, stepwise multiple regression analyses were conducted; with PA as the dependent variable at Early K and at End K. Table 3 presents the results.

**TABLE 3:** Prediction of PA in Early K and End of K from measures in Early K.

**Variable**	***R*^2^**	***R*^2^ change**	***F* change**
**PA – Early K**			
Letter naming	0.17	0.17	8.23^∗∗^
HTKS	0.29	0.11	6.22^∗∗^
**PA – End K**			
Letter naming	0.40	0.40	26.08^∗∗^

*^∗^*p* < 0.05; ^∗∗^*p* < 0.01. Early K = middle of kindergarten, December. End K = end of kindergarten, June. HTKS = Head–Toes–Knees–Shoulders.*

Letter naming at Early K was found to be a significant predictor of PA at Early K [*F*(1, 39) = 8.23, *p* < 0.001, *R*^2^ = 0.17, *R*^2^Adjusted = 0.17]. An additional contribution was observed for EF, which accounted for an extra 11% of the variance in PA [*F*(2, 38) = 6.28, *p* < 0.001, *R*^2^ = 0.9, *R*^2^Adjusted = 0.25]. Letter naming at Early K continued to be a significant predictor of PA at End K, accounting for 40% of the explained variance [*F*(1, 39) = 26.08, *p* < 0.001, *R*^2^ = 0.40, *R*^2^Adjusted = 0.38]. No other variables were significant predictors.

## Discussion

Phonological awareness is considered one of the crucial skills for literacy development among young children in different languages (e.g., Burgess and Lonigan, 1998; Aouad and Savage, 2009; Manolitsis and Tafa, 2011; Suortti and Lipponen, 2016), as indicated in a meta-analysis conducted by the National Early Literacy Panel (National Early Literacy Panel [NELP], 2008). The first aim of the current longitudinal study was to examine the development of PA skills in Hebrew, a transparent orthography at the middle and end of the kindergarten year. Overall, similar to previous studies in kindergarten children in English (e.g., Goswami and Bryant, 1990) and in transparent orthographies such as Greek (e.g., Manolitsis and Tafa, 2011), the results of the current study demonstrated rapid growth of PA skills among Hebrew-speaking children during the kindergarten year (Early K-End of K). This result emphasizes the importance of further examining the factors that may contribute to PA during this critical period.

The second goal of our study was to examine which PA skills develop at the middle and at the end of kindergarten in the Hebrew language. Most of the ten PA tasks showed that the phonological abilities of the children in the study improved significantly over this crucial period of time. Participants became better able to manipulate all units, including whole syllables, CV and phoneme syllables, and (consonantal) phonemes between the middle and the end of kindergarten. These results are consistent with previous studies conducted in other languages (e.g., Carroll et al., 2003; Shu et al., 2008). With regard to the developmental trajectories of the different skills, the present data is consistent with the English-language finding that syllable awareness precedes the development of phonemic awareness (e.g., Goswami and Bryant, 1990; Anthony and Francis, 2005). Alongside these findings, which appear to have universal applicability, other findings of the current study appear to be associated with unique characteristics of the Hebrew orthography. Children were better at deleting final consonants (CV/C) than they were at deleting initial consonants (C/VC). Thus, in accordance with Share and Blum (2005), in spoken Hebrew (as opposed to English) body-coda segmentation (CV + C) is more natural than onset-rime segmentation (C + VC). Share and Blum (2005) also proposed, based on previous studies showing the effects of Hebrew orthography on spoken language (Share and Levin, 1999; Ravid, 2005), that the body-coda preference is not merely orthographic but pre-orthographic, that is, purely phonological, and would also be found in children who had minimal exposure to the Hebrew writing system. The accessibility of CV units, as described above, facilitated the deletion of final consonants (in CVC words) and was apparent in the segmentation tasks as well. At first, the vast majority of responses (for both syllables and phonemes) began with a CV unit. Additionally, in the phoneme segmentation task, CV + V + VC segmentation decreased between the two time points, while CV + C segmentation increased. This probably demonstrates increasing access to phonemes because the V in the CV + V + CV is, according to Hebrew phonological theory, a CV in which the vowel is preceded by a glottal stop. This intriguing finding, which shows an increase in body-coda segmentation, may be due to children’s growing knowledge of the Hebrew writing system. The relationship between body-coda segmentation and literacy skills was also observed by Share and Blum (2005), who showed that kindergarten children’s preference for body-coda units increased alongside their awareness of Hebrew written language.

Returning to the phoneme segmentation task, despite the significant reduction of erroneous responses and the developing ability to segment CVC words into phonemes (C + V + C), this task continued to be the most difficult, resulting in a floor effect at both time points. It is assumed that the development of this specific ability only emerges once children have learned to read and write (Bentin et al., 1991), rather than developing naturally. Furthermore, similar to earlier work by Stahl and Murray (1994) that examined English-speaking kindergarten children, consonant deletion and synthesis were found to be easier than phoneme segmentation. The results of the current study appear to be associated with unique characteristics of the Hebrew orthography. This results are consistent with a group of studies demonstrating that PA develops as a function of the characteristics of the specific spoken language (i.e., Durgunoğlu and Öney, 1999).

To summarize, with regard to the first and second research questions and consistent with studies on English speakers, the current study demonstrated a rapid growth of PA skills among Hebrew-speaking children from the middle to the end of kindergarten. In addition, consistent with studies on English speakers, Hebrew-speaking children were significantly better at manipulations at the syllable level, as compared to phonemes or consonants. However, deletion of a final consonant was found to be easier for Hebrew-speaking children than deletion of an initial consonant. This finding emphasizes the *body-coda* segmentation tendency, which characterizes the Hebrew language structure and appears to be a language–specific feature.

Phonological awareness has been shown to have a reciprocal relationship with various measures, such as early literacy skills, and language and cognitive skills (e.g., Kirby et al., 2003; Michalczyk et al., 2013; Shaul and Schwartz, 2014). To explore and extend these findings with respect to the Hebrew language, the current study examined the relationships between PA and early literacy, language, and cognitive measures at the middle and end of kindergarten among Hebrew-speaking children, and attempted to determine whether these measures predict PA at the two time points. Moderate-strong positive correlations were found between PA and both letter naming and EF at Early K. A strong correlation between letter naming and PA was found at End K. This is in line with previous reports of a relationship between letter naming and PA among preliterate children in an English-speaking sample (Castles et al., 2009; Lerner and Lonigan, 2016), but contradicts results from a similar longitudinal study in a Greek (consistent orthography) kindergarten, which demonstrated via path analysis models that there was no direct link between earlier letter-sound and letter-name knowledge and subsequent PA by the end of kindergarten (Manolitsis and Tafa, 2011). An explanation for this relationship is that knowledge of letter names draws children’s attention to the phonemes represented by the letters. In Hebrew, all letter names begin with the represented phoneme (e.g., dalet for/d/), such that letter knowledge may indeed promote PA skills. In the current study, the significance of letter naming was supported by a regression analysis as well. Specifically, letter naming at Early K was found to be the most significant predictor of PA at End K, accounting for 40% of the explained variance. This highlights the significance of this literacy skill in PA development in Hebrew, and emphasizes the importance of learning letter names during kindergarten.

According to the current results, executive functioning should also be taken into account within the context of PA development. Alongside correlations with PA, regression analysis revealed that EF predicted PA at Early K. This is consistent with findings from previous studies showing predictive relationships between EF and PA among English-speaking preliterate children (Bierman et al., 2008; Welsh et al., 2010), and with the results of a study examining Hebrew-speaking kindergarten children (Shaul and Schwartz, 2014). In explaining this relationship, the authors of the latter study posited that substantial development in EF at this time of life helps facilitate pre-academic skills, and that similar abilities are involved in both EF and PA processes such as understanding of conventions and rules, and the ability to use them in different situations. It is important to note that in the current study, the strength of the relationship between EF and PA at End K was lower and not significant than the relationship between EF and PA at Early K. Thus, further studies should examine this variable and its relationship with PA during kindergarten.

Although naming speed was found in earlier studies to be positively correlated with PA (e.g., Schatschneider et al., 2002; Wolf et al., 2002), this was not the case in the current study. Rather, the current results support the proposition that naming speed and PA are separate factors (Bowers and Wolf, 1993; Manis et al., 1999), with PA more related to decoding accuracy, and naming speed to reading fluency and comprehension (Bowers, 1995; Cutting and Denckla, 2001; Kirby et al., 2003).

In the current study, vocabulary had the highest success rates of all the tasks administered. This might explain the lack of correlations between vocabulary and PA, as the former was characterized by low variability and the latter revealed great variability between participants. This finding, which is incompatible with those of prior studies (Metsala and Walley, 1998; Metsala, 1999; Ouellette and Haley, 2013; Hipfner-Boucher et al., 2014; Goodrich and Lonigan, 2015), also raises questions regarding the role of vocabulary knowledge in PA development at Early K. However, vocabulary production tasks yielding greater variance between children may lead to different results. In addition, it is possible that broader oral language skills, will demonstrate stronger relationships with PA.

With respect to working memory, in accordance with other studies (Alloway et al., 2004; Bandini et al., 2013), we expected to find a relationship with PA, presumably because phonological representations must be stored in memory when children are required to manipulate sounds, for example in phoneme segmentation and syllable deletion tasks (Alloway et al., 2004). However, we found no such relationship. It may be that the PA tasks employed did not require substantial working memory abilities, and that the inclusion of more complex manipulations involving a greater number of sound units would be required to reveal the relationship.

To summarize, the current study examined PA skills from a developmental perspective. In accordance with previous studies in various languages (Burgess and Lonigan, 1998; Lerner and Lonigan, 2016), we found significant relationships between letter identification skills and PA at the middle and end of kindergarten. The results also demonstrated the important relationship between PA and EF in kindergarten, adding to the growing body of work addressing this connection.

Several limitations of this study are worthy of mention. First, as a preliminary study, it was conducted on a small sample. Further research with larger sample sizes will likely enable extended conclusions and increased validity. Also, additional cognitive abilities, that may be related to PA were not included in the scope of this study, and should be examined in future work. Another limitation was the use of the same PA battery to test the participants at both time points. Future studies should use equivalent tasks for repeated testing.

Phonological awareness is a dynamic ability that varies significantly and develops in a short period of time during kindergarten. As the foundation of reading and writing development, it must be followed at this critical age, highlighting the need for studies that go beyond literacy skills and examine its relationship with a range of cognitive, language, and EF skills. The current findings also have important implications for intervention, as they provide information regarding the skills that should be strengthened in order to reinforce PA development in kindergarten. More generally, the study supports the idea that literacy practices, and preschool and kindergarten instruction and intervention, should take into account the importance of exposing children to letter knowledge that fosters PA.

The current study emphasizes the need for longitudinal research in the Hebrew language, to expand theoretical and practical knowledge of PA development in relation to cognitive, early literacy, and language skills, and in other languages, to learn more about processes that are universal and those that are unique to specific orthographies. In addition, it is important to continue examining the development of PA at early ages with relation to environmental factors (Reese et al., 2015), to better understand how to promote PA, and in designing interventions to support this crucial skill.

## Ethics Statement

This study was approved by the Research Ethics Committee of the Department of Education at the University of Haifa. All subjects gave written informed consent in accordance with the Declaration of Helsinki. The protocol was approved by the Ethical Committee of the Faculty of Education.

## Author Contributions

DW contributed to the data collection and statistical analysis as well as to writing the first draft and the development of the PA assessment tool. Both authors conceptualized this study, contributed to the writing and interpretation of the data, and agreed to be accountable for the content of the work.

## Conflict of Interest Statement

The authors declare that the research was conducted in the absence of any commercial or financial relationships that could be construed as a potential conflict of interest.
